# Plantar keratoderma of Sézary syndrome

**DOI:** 10.1002/ccr3.1168

**Published:** 2017-08-29

**Authors:** Konstantinos C. Fragkos

**Affiliations:** ^1^ University College London Hospitals London UK

**Keywords:** Cutaneous T‐cell lymphoma, plantar keratoderma, pruritus, Sézary syndrome

## Abstract

Sézary syndrome is an extremely rare form of cutaneous T‐cell lymphoma. It presents suddenly and is associated with a poor prognosis. Clinical recognition is crucial for the diagnostic process and initiation of appropriate treatment. Plantar keratoderma is usually pathognomonic for Sézary syndrome and clinicians should be alerted to its presence.

A 65‐year‐old woman presented with a 4‐year history of progressive refractory diffuse pruritus. She had been diagnosed 4 months earlier with cutaneous T‐cell lymphoma (CTCL) stage I (mycosis fungoides). After being treated unsuccessfully with PUVA for 31 sessions, she was eventually diagnosed with CTCL stage IVA_2_ (Sézary syndrome) on the basis of severe worsening pruritus, erythroderma, palmoplantar keratoderma with skin ulcerations causing cellulitis (Fig. 1A), left eye ectropion, lymphanedopathy, positive skin biopsy and left inguinal lymph node biopsy, raised lactate dehydrogenase, Sézary cells over 1000/*μ*L with positive clonality, CD4/CD8 ratio: 12.5, and CT chest, abdomen and pelvis showing inguinal and axillary lymphadenopathy with no visceral involvement. With bexarotene, interferon‐alfa, and extracorporeal photopheresis, her keratoderma stabilized (Fig. 1B), and after 2 months of treatment with keratolytics (6% salicylic acid in 70% propylene glycol and clobetasol), it largely cleared.

**Figure 1:**
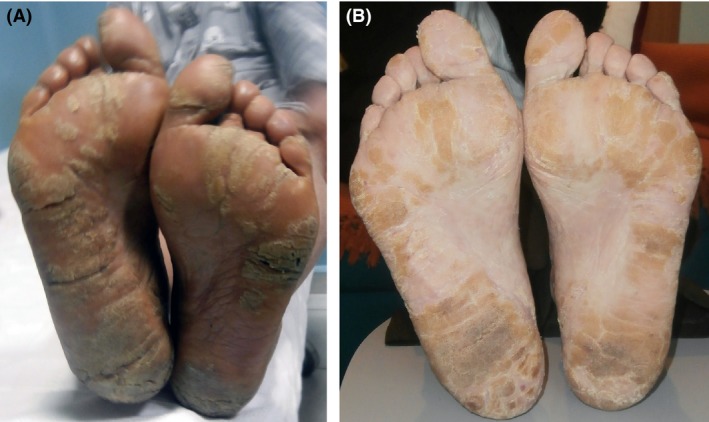
Plantar Keratoderma at the time of diagnosis (A) and after 1 month of treatment (B).

Sézary syndrome is CTCL stage IV and its incidence is 1 in 10,000,000 with predicted median survival between 2 and 4 years 1, 2. Its skin changes are characteristic and clinicians should be alarmed to severe pruritus with keratoderma as to exclude lymphoma. Despite the possibility to use first‐line agents as treatment, allogenic stem cell transplantation is considered the only curative approach.

## Informed Consent

Informed consent has been obtained for the publication of this clinical image.

## Authorship

KCF: acquired the images, wrote the manuscript, and has accountability for all aspects of the work.

## Conflict of Interest

None declared.
